# Retraction Note to: Inhibition of ZEB1-AS1 confers cisplatin sensitivity in breast cancer by promoting microRNA-129-5p-dependent ZEB1 downregulation

**DOI:** 10.1186/s12935-021-01759-5

**Published:** 2021-01-11

**Authors:** Jin Gao, Yuan Yuan, Lili Zhang, Shaorong Yu, Jianwei Lu, Jifeng Feng, Sainan Hu

**Affiliations:** Department of Medical Oncology, Jiangsu Cancer Hospital, Jiangsu Institute of Cancer Research, The Affiliated Cancer Hospital of Nanjing, Medical University, No. 42 Baiziting, Nanjing, 210009 Jiangsu People’s Republic of China

## Retraction to: Cancer Cell Int (2020) 20:90 10.1186/s12935-020-1164-8

The Editors-in-Chief have retracted this article [1] at the request of the authors because concerns have been raised regarding validity of data.

The authors informed the journal that the bioinformatics analysis and all laboratory contents had been performed by an external company. The outsourced laboratory content includes cell culture, FISH, dual luciferase reporter gene assay, RNA pull down assay, RNA immunoprecipitation (RNA IP) assay, cell grouping and transfection, RT qPCR, western blot analysis, chemosensitivity assay and flow cytometry. This was not made clear in this article. The company has not responded to requests from authors to share raw data. Consequently, the data cannot be verified.

The authors stated that they completed the rest of the work, including clinical patient samples immunohistochemistry analysis, statistical analysis and discussion.

All authors agree to this retraction.
